# Genetic and biological characterisation of an avian-like H1N2 swine influenza virus generated by reassortment of circulating avian-like H1N1 and H3N2 subtypes in Denmark

**DOI:** 10.1186/1743-422X-10-290

**Published:** 2013-09-18

**Authors:** Ramona Trebbien, Karoline Bragstad, Lars Erik Larsen, Jens Nielsen, Anette Bøtner, Peter MH Heegaard, Anders Fomsgaard, Birgitte Viuff, Charlotte Kristiane Hjulsager

**Affiliations:** 1 National Veterinary Institute, Technical University of Denmark, Bülowsvej 27, Frederiksberg C, DK-1870, Denmark; 2 Statens Serum Institut, Artillerivej 5, Copenhagen S, DK-2300, Denmark; 3 National Veterinary Institute, Technical University of Denmark, Lindholm Ø, Kalvehave, DK-4771, Denmark; 4 Department of Veterinary Disease Biology, Faculty of Health and Medical Sciences, University of Copenhagen, Dyrlægevej 88, Frederiksberg C, 1870, Denmark

**Keywords:** Influenza virus, Swine, H1N2 subtype, Reassortment, Phylogeny, Infection

## Abstract

**Background:**

The influenza A virus subtypes H1N1, H1N2 and H3N2 are the most prevalent subtypes in swine. In 2003, a reassorted H1N2 swine influenza virus (SIV) subtype appeared and became prevalent in Denmark. In the present study, the reassortant H1N2 subtype was characterised genetically and the infection dynamics compared to an “avian-like” H1N1 virus by an experimental infection study.

**Methods:**

Sequence analyses were performed of the H1N2 virus. Two groups of pigs were inoculated with the reassortant H1N2 virus and an “avian-like” H1N1 virus, respectively, followed by inoculation with the opposite subtype four weeks later. Measurements of HI antibodies and acute phase proteins were performed. Nasal virus excretion and virus load in lungs were determined by real-time RT-PCR.

**Results:**

The phylogenetic analysis revealed that the reassorted H1N2 virus contained a European “avian-like” H1-gene and a European “swine-like” N2-gene, thus being genetically distinct from most H1N2 viruses circulating in Europe, but similar to viruses reported in 2009/2010 in Sweden and Italy. Sequence analyses of the internal genes revealed that the reassortment probably arose between circulating Danish “avian-like” H1N1 and H3N2 SIVs. Infected pigs developed cross-reactive antibodies, and increased levels of acute phase proteins after inoculations. Pigs inoculated with H1N2 exhibited nasal virus excretion for seven days, peaking day 1 after inoculation two days earlier than H1N1 infected pigs and at a six times higher level. The difference, however, was not statistically significant. Pigs euthanized on day 4 after inoculation, had a high virus load in all lung lobes. After the second inoculation, the nasal virus excretion was minimal. There were no clinical sign except elevated body temperature under the experimental conditions.

**Conclusions:**

The “avian-like” H1N2 subtype, which has been established in the Danish pig population at least since 2003, is a reassortant between circulating swine “avian-like” H1N1 and H3N2. The Danish H1N2 has an “avian-like” H1 and differs from most other reported H1N2 viruses in Europe and North America/Asia, which have H1-genes of human or “classical-swine” origin, respectively. The variant seems, however, also to be circulating in countries like Sweden and Italy. The infection dynamics of the reassorted “avian-like” H1N2 is similar to the older “avian-like” H1N1 subtype.

## Introduction

Influenza A virus is a common cause of respiratory disease in swine. The virus also infects other species including humans and birds. Influenza A viruses are classified into different subtypes based on the surface glycoproteins hemagglutinin (HA) and neuraminidase (NA). So far, 17 different HA and 10 different NA subtypes have been described [1-4]. The predominant swine influenza virus (SIV) subtypes worldwide are H1N1, H1N2 and H3N2, all of which show considerable diversity within each subtype. Until the emergence of the pandemic influenza A (H1N1) 2009 (H1N1pdm09) virus, which possesses a “classical-swine” H1 HA gene, the European H1N1 SIV belonged exclusively to the “avian-like” lineage of SIV with all eight gene segments of avian origin [5] or they were reassortants hereof retaining the “avian-like” H1 HA. European H3N2 SIV are human-avian reassortants retaining the HA and NA genes from human H3N2 virus, whereas the remaining gene segments originate from “avian-like” H1N1 SIV [6]. H1N2, which is the most recently recognized new European SIV subtype, was first reported from Great Britain in 1995 [7]. This virus possessed an HA closely related to that of human A/England/80-like H1N1 viruses, an NA probably derived from a swine H3N2 virus, and internal gene segments of avian origin [8]. Subsequent spread of related “human-like” H1N2 viruses has been reported from several European countries (Italy and France [9], Belgium [10], Germany [11] and Spain [12]) including reassortments containing HA from the “human-like” H1N2 [13]. “Human-like” H1N2 viruses (so-called delta clade viruses) have also been reported from North America [14] but other H1N2 viruses that are circulating in North America and Asia [15-19] contain HA genes of “classical-swine” H1N1 origin descending from the very first SIVs, which were isolated from pigs in USA in 1930 [20] and resemble human viruses responsible for the 1918 Spanish flu pandemic. Since the emergence of the H1N1pdm09 virus in swine, several descendant reassortant viruses have been recognized, including H1N2 with all genes from the pandemic virus except the N2 [21] and an H1N2 with HA and NA from the “human-like” swine H1N2 and the rest of the genes from H1N1pdm09 virus [22]. H1N2 viruses with “avian-like” H1 have earlier been reported from France and Italy [9,23], but without having been established in the regional pig populations. In Denmark, the H1N2 subtype was first recognized in a lung tissue sample from a pig with coughing, fever and panting, showing indications of bronchopneumonia. This pig was submitted to the National Veterinary Institute for diagnosis of SIV in 2003. The detected virus (A/swine/Denmark/12687/2003(H1N2) had an “avian-like” H1, and this reassorted H1N2 subtype has since then continuously been detected from lung tissue and nasal swabs from Danish pigs throughout the country, and is now established in Denmark (unpublished data). Prior to 2003, only the H1N1 and H3N2 subtypes had been isolated in Denmark [24]. The “avian-like” H1N2 has been reported previously from Sweden and Italy [25-27]. The aim of the present study was to make a genetic characterization of the Danish reassorted “avian-like” H1N2 virus and to compare the cross-protection and infection dynamics of the reassorted “avian-like” H1N2 with the older endemic “avian-like” H1N1 virus.

## Results

### Analysis of sequences and phylogenetics

**Figure 1:**
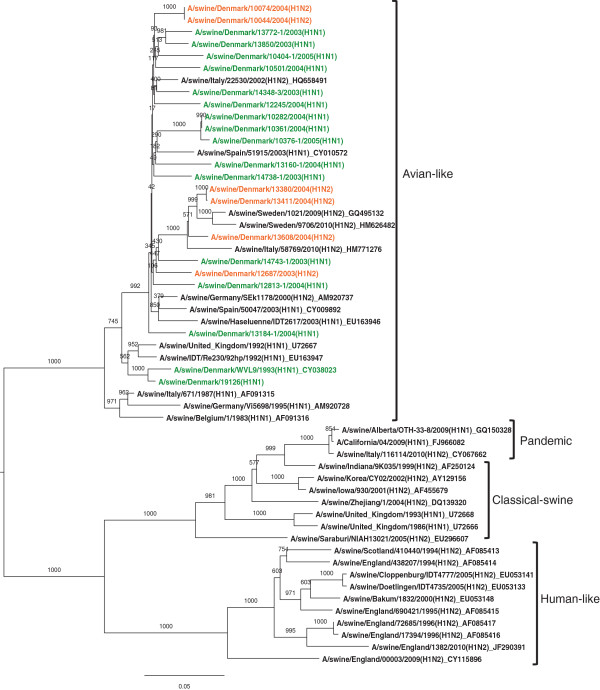
**Phylogenetic analysis of the HA nucleotide sequences of Danish H1N2 viruses.** The tree shows the phylogenetic relationship of the HA gene (position 1–1701) from the reassorted Danish H1N2 swine influenza virus to representative HA genes of H1 swine origin. Gene sequences were aligned with Muscle using CLC DNA Workbench version 6.8.1. Neighbour Joining trees were calculated with a bootstrap value of 1000. Danish H1N2 sequences are in orange and Danish H1N1 sequences are in green.

**Figure 2:**
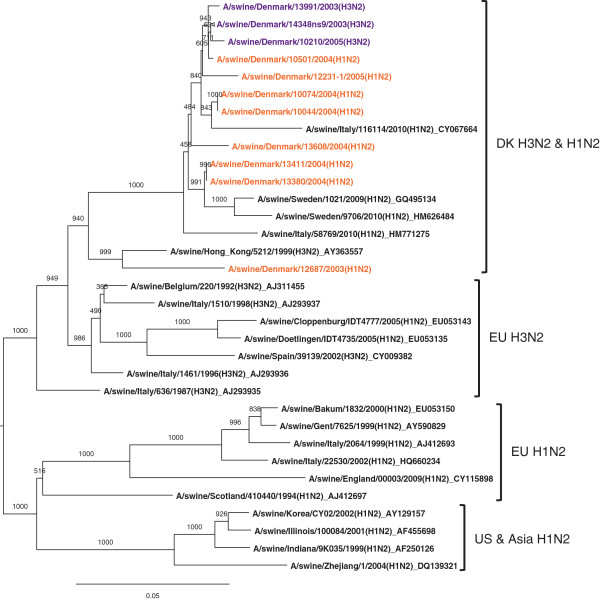
**Phylogenetic analysis of the NA nucleotide sequences of Danish H1N2 viruses.** The tree shows the phylogenetic relationship of the NA gene (position 22–1380) from the reassorted Danish H1N2 swine influenza virus to representative NA genes of N2 swine origin. Gene sequences were aligned with Muscle using CLC DNA Workbench version 6.8.1. Neighbour Joining trees were calculated with a bootstrap value of 1000. Danish H1N2 sequences are in orange and Danish H3N2 sequences are in purple.

**Figure 3:**
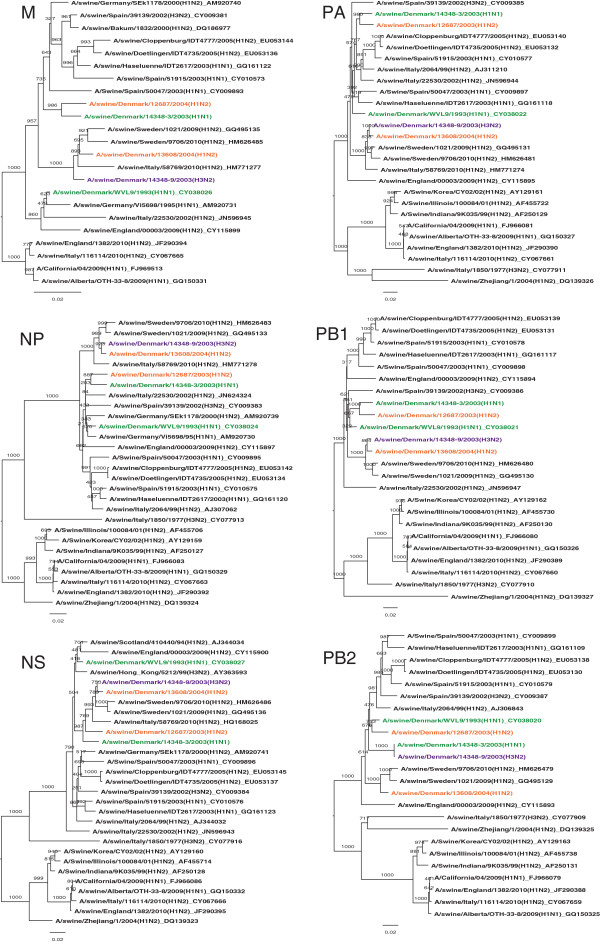
**Phylogenetic analysis of the internal genes of Danish H1N2 viruses.** The trees show the phylogenetic relationship of the reassorted Danish H1N2 swine influenza virus to representative Danish SIV and reference nucleotide sequences for the M-gene (position 1–971), NP-gene (position 1–1497), NS-gene (position 1–838), PA-gene (position 1–2151), PB1-gene (position 1–2267), and PB2-gene (position 709–2119). Gene sequences were aligned with Muscle using CLC DNA Workbench version 6.8.1. Neighbour Joining trees were calculated with a bootstrap value of 1000. Danish H1N2 sequences are in orange, Danish H1N1 sequences are in green, and Danish H3N2 sequences are in purple.

Viruses included in the study are listed in Table 1 with GenBank accession numbers. The phylogenetic analysis at the nucleotide level showed that the HA of the Danish H1N2 SIV´s was closely related to the HA gene of concurrently circulating Danish H1N1 viruses, whereas the NA gene was closely related to that of concurrent H3N2 viruses (Figures 1 and 2). The HA gene was of the avian-like lineage and the identity to the concurrent Danish H1N1 viruses was 93–96% (Table 2). The identity of the HA gene between the Danish H1N2 viruses and the reference virus A/swine/Scotland/410440/1994(H1N2) as well as contemporary European human-like H1N2 viruses from Germany were ~73%. The NA gene of the Danish H1N2 viruses clustered phylogenetically with concurrent Danish H3N2 viruses with an identity between 91–99% (Table 2). The nucleotide sequence comparisons of all gene segments are shown in Table 3 and phylogenetic trees for the internal genes are shown in Figure 3. The nucleotide sequences of the internal genes of two H1N2 isolates were determined and they showed a high degree of identity with those of either the H1N1 and/or H3N2 Danish contemporary SIV isolates (93–99% nucleotide identity). The comparisons also revealed that A/swine/Denmark/13608/2004(H1N2) had more genes with high identity to A/swine/Denmark/14348-9/2003(H3N2) than to A/swine/Denmark/14348-3/2003(H1N1), whereas A/swine/Denmark/12687/2003(H1N2) was more identical to A/swine/Denmark/14348-3/2003(H1N1). The phylogenetic analyses (Figure 1, 2, and 3) supported two clusters represented by A/swine/Denmark/13608/2004(H1N2) and A/swine/Denmark/12687/2003(H1N2), respectively. In general the internal genes of the Danish reassortant H1N2 viruses clustered phylogenetically with European viruses and separated from American “classical-swine” H1 and H1pdm09 virus strains. In all gene segments, A/swine/Denmark/13608/2004(H1N2) had a very high nucleotide identity (HA gene ~96%, NA gene ~96%) to H1N2 reassortant viruses detected in Sweden and Italy in 2009 and 2010 (Table 2) containing avian-like H1 and N2 from H3N2 SIV, and they group closely together in the phylogenetic trees. At the amino acid level, seven N-glycosylation sites (positions 28, 40, 71, 179, 291, 498, 557) were predicted for A/swine/Denmark/13608/2004(H1N2) in the HA protein, whereas only 4 sites (positions 28, 40, 498, 557) were predicted in A/swine/Denmark/12687/2003(H1N2). The Italian A/swine Italy/58769/2010(H1N2) had conserved glycosylation sites similar to A/swine/Denmark/13608/2004(H1N2), but had an additional predicted site at position 212. The Swedish viruses showed all the predicted glycosylation sites of the A/swine/Denmark/13608/2004(H1N2) virus except for 71 or 179 in 2010 and 2009, respectively. In the receptor binding domain of the HA protein, all Danish H1 viruses as well as the Swedish and Italian viruses had a D in position 190. In position 225, A/swine/Denmark/13608/2004(H1N2) and the Italian virus had K, whereas the A/swine/Denmark/12687/2003(H1N2) had an E. The Swedish viruses had a 225 T motif similar to the Danish A/swine/Denmark/13411/2004(H1N2) and A/swine/Denmark/13880/2004(H1N2) viruses. The NA sequences of the Danish H1N2 viruses showed no indication of resistance to NA-inhibitors, whereas all the Danish M sequences deduced resistance against amantadine.

**Table 1:** Viruses included in the study

***Virus***	***Subtype***	***Passage history***	***Sample material***	***GenBank accession no.***
A/swine/Denmark/12231-1/2005(H1N2)	H1N2	none	Lung	KC900232
A/swine/Denmark/10074/2004(H1N2)	H1N2	3MDCK	Lung	KC900233, KC900234
A/swine/Denmark/10044/2004(H1N2)	H1N2	2MDCK	Lung	KC900235, KC900236
A/swine/Denmark/10501//2004(H1N2)	H1N2	4MDCK	Lung	KC900237, KC900238
A/swine/Denmark/13411/2004(H1N2)	H1N2	2SK	Lung	KC900249, KC900250
A/swine/Denmark/13380/2004(H1N2)	H1N2	1SK	Lung	KC900251, KC900252
A/swine/Denmark/13608/2004(H1N2)	H1N2	1SK	Lung	KC900253 to KC900260
A/swine/Denmark/12687/2003(H1N2)	H1N2	4SK	Lung	KC900261 to KC900268
A/swine/Denmark/13991/2003(H3N2)	H3N2	3SK	Lung	KC900239
A/swine/Denmark/10210/2005(H3N2)	H3N2	1SK	Lung	KC900240
A/swine/Denmark/14348-9/2003(H3N2)	H3N2	1SK	NS	KC900241 to KC900248
A/swine/Denmark/14738-1/2003(H1N1)	H1N1	4MDCK	Lung	KC900269
A/swine/Denmark/13184-1/2004(H1N1)	H1N1	1SK	Lung	KC900270
A/swine/Denmark/12813-1/2004(H1N1)	H1N1	1SK	Lung	KC900271
A/swine/Denmark/14348-3/2003(H1N1)	H1N1	1SK	Lung	KC900272 to KC900279
A/swine/Denmark/12245/2004(H1N1)	H1N1	1SK	Lung	KC900280
A/swine/Denmark/10404-1/2005(H1N1)	H1N1	1SK	Lung	KC900281
A/swine/Denmark/13772-1/2003(H1N1)	H1N1	2SK	Lung	KC900282
A/swine/Denmark/13850/2003(H1N1)	H1N1	2SK	Lung	KC900283
A/swine/Denmark/13160-1/2004(H1N1)	H1N1	2SK	Lung	KC900284
A/swine/Denmark/10376-1/2005(H1N1)	H1N1	2SK	Lung	KC900285
A/swine/Denmark/10282/2004(H1N1)	H1N1	2MDCK	Lung	KC900286
A/swine/Denmark/10361/2004(H1N1)	H1N1	4MDCK	Lung	KC900287
A/swine/Denmark/14743-1/2003(H1N1)	H1N1	3SK	Lung	KC900288
A/swine/Denmark/19126/1993(H1N1)	H1N1	3SK	Lung	KC900289

Viruses included in the study with accession numbers for sequences submitted to Genbank. *MDCK*; Madin-Darby canine kidney cells, *SK*; primary swine kidney cells, *NS*; nose swab.

**Table 2:** Percent identity at the nucleotide level for Danish SIV viruses and reference viruses

**Virus**		**1**	**2**	**3**	**4**	**5**	**6**	**7**	**8**	**9**	**10**	**11**	**12**	**13**	**14**	**15**	**16**	**17**	**18**	**19**	**20**	**21**	**22**	**23**
A/swine/Denmark/13608/2004(H1N2)	1		98	98	95	95	95	96	97	74	96	74	74	74	93	94	95	94	73	n.a.	n.a.	n.a.	n.a.	n.a.
A/swine/Denmark/13411/2004(H1N2)	2	98		100	95	95	95	97	98	73	96	74	73	74	93	94	94	94	73	n.a.	n.a.	n.a.	n.a.	n.a.
A/swine/Denmark/13380/2004(H1N2)	3	98	100		95	95	95	97	98	73	96	74	73	74	93	94	95	94	73	n.a.	n.a.	n.a.	n.a.	n.a.
A/swine/Denmark/12687/2003(H1N2)	4	92	92	92		96	96	93	94	73	93	74	73	74	94	94	95	95	73	n.a.	n.a.	n.a.	n.a.	n.a.
A/swine/Denmark/10044/2004(H1N2)	5	98	99	99	92		100	93	93	74	93	74	73	74	94	95	95	95	74	n.a.	n.a.	n.a.	n.a.	n.a.
A/swine/Denmark/10074/2004(H1N2)	6	98	99	99	92	100		93	93	74	93	74	73	74	94	95	95	95	74	n.a.	n.a.	n.a.	n.a.	n.a.
A/swine/Sweden/9706/2010(H1N2)_HM626482	7	96	98	98	90	96	96		98	73	94	73	72	74	91	92	93	92	73	n.a.	n.a.	n.a.	n.a.	n.a.
A/swine/Sweden/1021/2009(H1N2)_GQ495132	8	97	98	98	90	97	97	98		73	95	74	73	74	92	93	93	93	73	n.a.	n.a.	n.a.	n.a.	n.a.
A/swine/Italy/116114/2010(H1N2)_CY067662	9	95	96	96	90	97	97	94	94		73	77	76	95	74	73	73	74	99	n.a.	n.a.	n.a.	n.a.	n.a.
A/swine/Italy/58769/2010(H1N2)_HM771276	10	96	97	97	91	97	97	95	96	94		74	74	74	91	92	93	93	73	n.a.	n.a.	n.a.	n.a.	n.a.
A/swine/Scotland/410440/1994(H1N2)_AF085413	11	88	89	89	88	88	88	88	88	87	87		92	78	74	73	74	73	77	n.a.	n.a.	n.a.	n.a.	n.a.
A/swine/Doetlingen/IDT4735/2005(H1N2)_EU053133	12	90	90	90	90	90	90	89	89	88	89	87		77	73	73	73	73	76	n.a.	n.a.	n.a.	n.a.	n.a.
A/swine/Indiana/9 K035/1999(H1N2)_AF250124	13	86	87	87	86	87	87	86	86	85	86	89	86		75	74	74	74	95	n.a.	n.a.	n.a.	n.a.	n.a.
A/swine/Denmark/19126/1993(H1N1)	14	n.a.	n.a.	n.a.	n.a.	n.a.	n.a.	n.a.	n.a.	n.a.	n.a.	n.a.	n.a.	n.a.		93	94	93	74	n.a.	n.a.	n.a.	n.a.	n.a.
A/swine/Denmark/12245/2004(H1N1)	15	n.a.	n.a.	n.a.	n.a.	n.a.	n.a.	n.a.	n.a.	n.a.	n.a.	n.a.	n.a.	n.a.	n.a.		94	95	73	n.a.	n.a.	n.a.	n.a.	n.a.
A/swine/Denmark/14743-1/2003(H1N1)	16	n.a.	n.a.	n.a.	n.a.	n.a.	n.a.	n.a.	n.a.	n.a.	n.a.	n.a.	n.a.	n.a.	n.a.	n.a.		95	73	n.a.	n.a.	n.a.	n.a.	n.a.
A/swine/Denmark/12813-1/2004(H1N1)	17	n.a.	n.a.	n.a.	n.a.	n.a.	n.a.	n.a.	n.a.	n.a.	n.a.	n.a.	n.a.	n.a.	n.a.	n.a.	n.a.		74	n.a.	n.a.	n.a.	n.a.	n.a.
A/swine/Alberta/OTH-33-8/2009(H1N1pdm)_GQ150328	18	n.a.	n.a.	n.a.	n.a.	n.a.	n.a.	n.a.	n.a.	n.a.	n.a.	n.a.	n.a.	n.a.	n.a.	n.a.	n.a.	n.a.		n.a.	n.a.	n.a.	n.a.	n.a.
A/swine/Denmark/13991/2003(H3N2)	19	98	98	98	92	99	99	96	97	96	96	89	90	87	n.a.	n.a.	n.a.	n.a.	n.a.		n.a.	n.a.	n.a.	n.a.
A/swine/Denmark/10210/2005(H3N2)	20	98	98	98	91	99	99	96	97	96	96	88	90	86	n.a.	n.a.	n.a.	n.a.	n.a.	99		n.a.	n.a.	n.a.
A/swine/Denmark/14348 ns9/2003(H3N2)	21	98	99	99	92	99	99	96	97	96	97	89	90	87	n.a.	n.a.	n.a.	n.a.	n.a.	99	99		n.a.	n.a.
A/swine/Spain/39139/2002(H3N2)_CY009382	22	90	91	91	91	90	90	89	89	88	89	87	94	85	n.a.	n.a.	n.a.	n.a.	n.a.	90	90	90		n.a.
A/swine/Hong Kong/5212/1999(H3N2)_AY363557	23	93	94	94	95	93	93	92	92	91	92	89	92	87	n.a.	n.a.	n.a.	n.a.	n.a.	93	93	94	92	

Percent identity at the nucleotide level for Danish SIV viruses and reference SIV viruses calculated for the HA and NA gene. Lower part of the table is between N2 sequences and upper part of the table is between H1 sequences, n.a.; not applicable.

**Table 3:** Percentage nucleotide identity between Danish subtypes for all 8 influenza A segments

***Gene segment***	***H1N2-13608 vs.***		***H1N2-12687 vs.***		***H1N2- 13608 vs.***	***Region compared***
	**H1N1**	**H3N2**	**H1N1**	**H3N2**	**H1N2-12687**	
PB2	93	93	94	94	95	709-2119
PB1	93	**99**	**96**	93	93	1-2267
PA	94	**99**	**96**	95	94	1-2151
HA	**94**	51	**95**	50	95	1-1701
NP	93	**99**	**96**	93	94	1-1497
NA	49	**98**	49	**92**	92	22-1380
M	96	**99**	**98**	96	96	1-971
NS	96	**99**	96	96	96	1-838

The percentage identity of nucleotide sequences were calculated with CLC DNA Workbench version 6.8.1. Highest matches are in bold.

H1N1: A/Swine/Denmark/14348lu3/2003(H1N1); H3N2: A/Swine/Denmark/14348 ns9/2003(H3N2); H1N2-12687: A/Swine/Denmark/12687/2003(H1N2); H1N2-13608: A/Swine/Denmark/13608/2004(H1N2).

### Clinical signs and gross pathology

**Figure 4:**
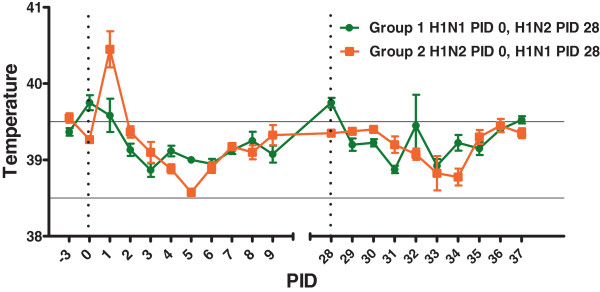
**Body temperature measurements.** Body temperature measurements after first inoculation post inoculation day (PID) 0 and second inoculation PID 28 for both group 1 and 2 pigs. The stapled lines indicates respectively first and second inoculation.

There were few visible clinical signs during the experiment in both groups. After the first inoculation at post inoculation day (PID) 0, an increase in body temperature seen on PID 1 in most of the pigs inoculated with H1N2 returned to normal level on PID 2 (Figure 4). After the second inoculation on PID 28, elevated body temperatures could not be observed (Figure 4).

Pigs inoculated with H1N1 on PID 0 and euthanized on day 4 (group 1) had consolidated lobules in the cranial lung lobes. In the left cranial lobe and middle lobe, the consolidated areas constituted <10% of the lobes. H1N2 inoculated pigs euthanized on PID 4 (group 2), had more widespread consolidations in the lung lobes compared to the pigs in group 1. In the left cranial lobes, approximately 80% of the tissue was consolidated, the middle lobes were approximately 40% consolidated and the accessory lobes were 20% consolidated. In the right and left caudal lobes, consolidated areas were only evident in the most cranial part and only a few lobules were consolidated in the right cranial lobe. The pigs euthanized PID 56 had no gross-pathological changes.

### Acute phase protein measurements

**Figure 5:**
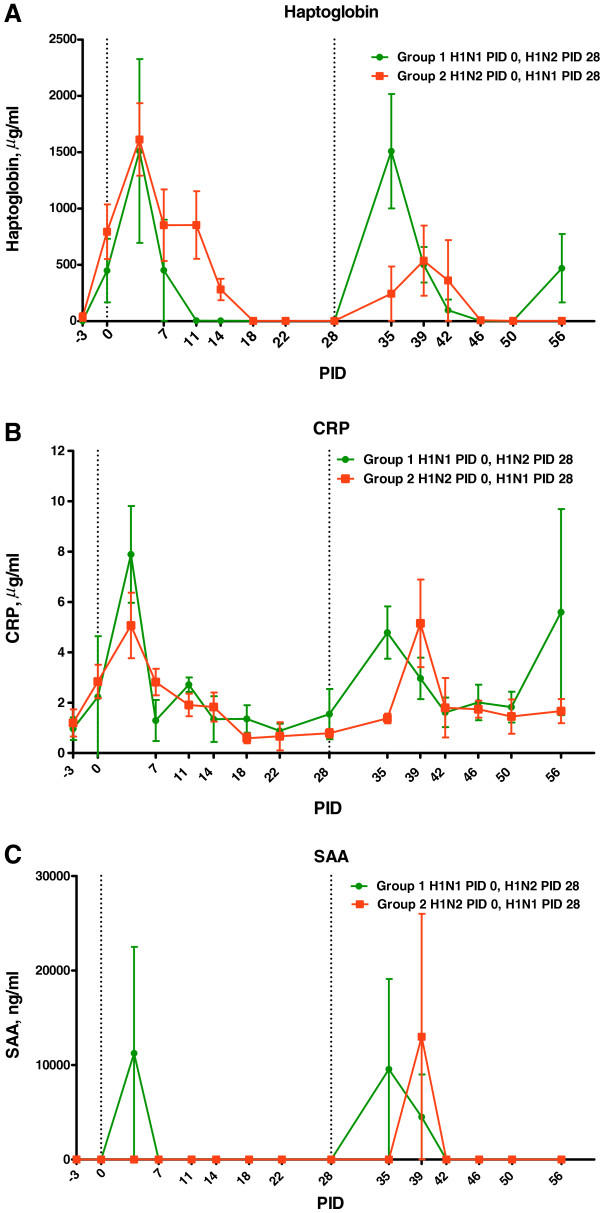
**Acute phase protein levels in serum.** The graphs show the acute phase protein levels in the serum samples from group 1 and 2 pigs, the stapled lines indicates first and second inoculation, respectively. **A**: Haptoglobin, **B**: C - Reactive Protein (CRP), **C**: Serum Amyloid A (SAA). PID: post inoculation day.

The acute phase responses were included as an objective measurement of infection severity. Results from measurements of the acute phase protein C - Reactive Protein (CRP), Serum Amyloid A (SAA) and haptoglobin are shown in Figure 5. Both groups of pigs (group 1: H1N1 followed by H1N2, group 2: H1N2 followed by H1N1) showed haptoglobin and CRP responses following each of the inoculations, peaking on PID 4 and 35 (group 1) or 4 and 39 (group 2). No SAA response was seen for group 2 after the H1N2 infection, however, these pigs did show an SAA response after the subsequent H1N1 inoculation. In contrast, an SAA response was seen both at the first infection (H1N1) and the second infection (H1N2) for group 1. The difference between groups for all three acute phase proteins were not statistically significant when using an unpaired *t*-test with 95% confidence interval (CRP, p = 0,4; haptoglobin, p = 0,8; SAA, p = 0,5).

### Antibody detection

**Figure 6:**
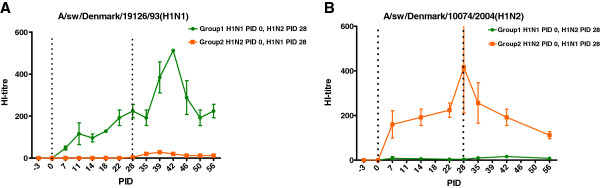
**Antibody levels in serum. A**: Results from the HI-test on serum samples from the experiment using A/swine/Denmark/19126/1993 (H1N1) as antigen. **B**: Results from the HI-test using A/swine/Denmark/10074/2004 (H1N2) as antigen. The stapled lines indicate day of inoculation respectively post infection day (PID) 0 and PID 28.

The results of the HI-test after 1^st^ and 2^nd^ inoculations are shown in Figure 6. For both groups of pigs an antibody response against the homologue HI-test antigen was elicited after the first inoculation and after second inoculation with the other virus (heterologous to the HI-test antigen) a boost in the antibody response was seen for group 1 pigs. This was not clearly observed for group 2 pigs. In contrast, no or very low titres of HI antibodies were seen after the first inoculation when the heterologous virus was used as antigen, however after the second inoculations, antibody responses were seen in both groups.

### Measurement of nasal virus excretion and virus load in the lung by real time RT-PCR

The results of the real time RT-PCR screening are shown for the nasal swab samples in Table 4. In general, influenza A virus RNA was detected in nasal swabs in both groups until day 7 after the 1^st^ inoculation. After the 2^nd^ inoculation on day 28, the pigs were negative by this assay, except for two pigs in group 2 that were positive on day 29 and 35 and only day 35, respectively. Virus was detected with the screening assay in all nine lung samples from pigs euthanized on day 4 but not from pigs euthanized on day 56.

**Table 4:** Results from the real time RT-PCR screenings assay detecting the NP-gene on nasal swabs

***PID***	***Group1***						***Group2***					
	**1**	**2**	**3**	**4**	**5**	**6**	**7**	**8**	**9**	**10**	**11**	**12**
-3	-	-	-	-	-	-	-	-	-	-	-	-
0	-	-	-	-	-	-	-	-	-	-	-	-
1	+	+	+	+	+	+	+	+	+	+	+	+
2	+	+	+	+	+	+	+	+	+	+	+	+
3	+	+	+	+	+	+	+	+	+	+	+	+
4	+	+	+	+	+	+	+	+	+	+	+	+
5	n.a.	n.a.	+	+	+	+	n.a.	n.a.	+	+	+	+
6	n.a.	n.a.	+	+	+	+	n.a.	n.a.	+	+	+	+
7	n.a.	n.a.	+	+	(+)´	-	n.a.	n.a.	+	(+)´	+	(+)´
8	n.a.	n.a.	-	-	-	-	n.a.	n.a.	-	(+)´	-	-
9,11,14,18,22	n.a.	n.a.	-	-	-	-	n.a.	n.a.	-	-	-	-
28	n.a.	n.a.	-	-	-	-	n.a.	n.a.	-	-	-	-
29	n.a.	n.a.	-	-	-	-	n.a.	n.a.	-	-	-	+
30,31,32,33,34	n.a.	n.a.	-	-	-	-	n.a.	n.a.	-	-	-	-
35	n.a.	n.a.	-	-	-	-	n.a.	n.a.	-	-	+	+
36,37,39,42,46,50,56	n.a.	n.a.	-	-	-	-	n.a.	n.a.	-	-	-	-

Group 1 pigs (1–6) were inoculated post inoculation (PID) 0 with A/swine/Denmark/19126/1993 (H1N1) and reinoculated PID 28 with A/swine/Denmark/10074/2004 (H1N2). Group 2 pigs (7–12) were inoculated post inoculation (PID) 0 with A/swine/Denmark/10074/2004 (H1N2) and reinoculated PID 28 with A/swine/Denmark/19126/1993 (H1N1). +: Positive samples; -: negative samples; n.a.: not applied as pigs were euthanized PID 4; (+)´: in double testing one had a positive ct-value.

**Figure 7:**
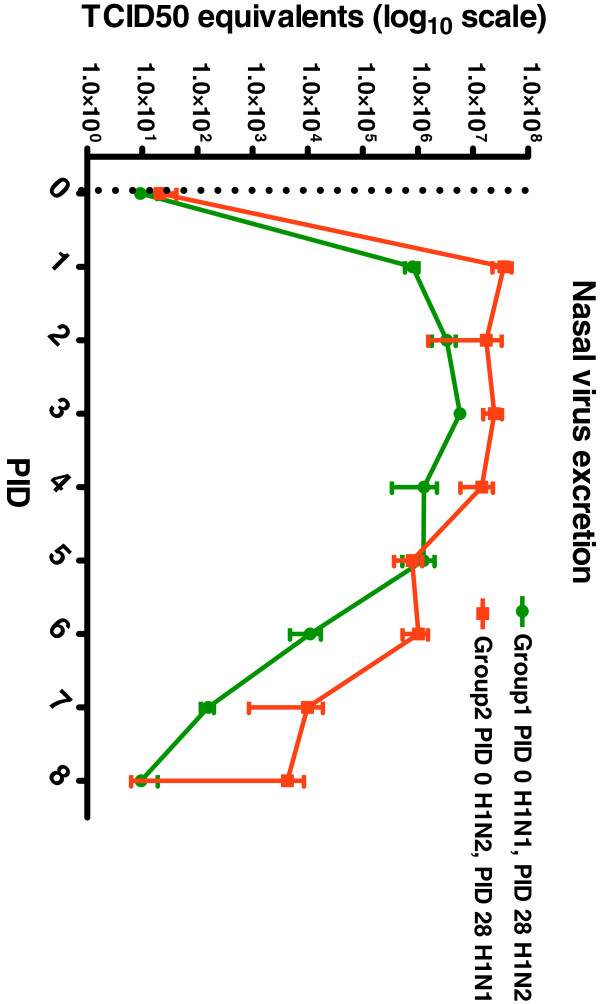
**Nasal virus excretion.** Nasal virus excretion measured with quantitative real time RT-PCR detecting the matrix gene. Group 1 pigs were inoculated post inoculation day (PID) 0 with H1N1 and group 2 pigs were inoculated PID 0 with H1N2 (stapled line).

The results of the quantitative real time RT-PCR assay on the positive samples showed virus excretions from PID 1 to PID 6–8 (Figure 7). For the pigs in group 1 (H1N1 inoculated), the virus titre peaked with an average of 5.8 × 10^6^ TCID50 equivalents on PID 3. For the pigs in group 2 (H1N2 inoculated), the peak was approximately 6 times higher reaching 3.6 × 10^7^ TCID50 equivalents PID 1. In group 2, two nasal swabs from one pig were found to be positive with a virus load of 5.6 × 10^2^ TCID50 equivalents on PID 29 and 1.9 × 10^2^ TCID50 equivalents on PID 35. A second pig in group 2 was positive on PID 35 with a titre of 1.1 × 10^3^ TCID50 equivalents. The difference in nasal virus excretion between groups was not significant with a p-value at 0.06 in unpaired *t*-test with a 95% confidence interval. Virus load in the lungs of pigs euthanized on PID 4 varied between 10^4^ and 10^9^ TCID50 equivalents in the different lung sections (Table 5). Furthermore, the amount of virus in the different areas of the lungs varied between the pigs. The average virus load of the nine lung sections for pigs in groups 1 and 2 were 1.77 × 10^8^ TCID50 equivalents and 2.58 × 10^7^ TCID50 equivalents, respectively. Thus, the virus load was about 7-fold higher in the group of H1N1 infected pigs compared to the H1N2 infected pigs, however this difference was not significant (p = 0.06).

**Table 5:** Virus load in lung samples

***Lung sections***	***Group 1***		***Group 2***	
	**Pig 1**	**Pig 2**	**Pig 7**	**Pig 8**
Lu1	6.0 × 10^7^	9.5 × 10^6^	1.5 × 10^5^	2.3 × 10^7^
Lu2	7.1 × 10^6^	1.4 × 10^9^	3.1 × 10^7^	1.8 × 10^8^
Lu3	1.1 × 10^7^	5.2 × 10^8^	7.5 × 10^6^	3.7 × 10^7^
Lu4	2.0 × 10^5^	7.1 × 10^7^	3.2 × 10^4^	4.5 × 10^4^
Lu5	3.5 × 10^8^	1.8 × 10^8^	7.6 × 10^7^	1.4 × 10^5^
Lu6	3.8 × 10^6^	1.5 × 10^7^	6.2 × 10^6^	2.1 × 10^5^
Lu7	7.8 × 10^6^	2.1 × 10^8^	3.7 × 10^7^	3.2 × 10^7^
Lu8	9.8 × 10^7^	4.8 × 10^7^	7.1 × 10^6^	2.9 × 10^7^
Lu9	1.6 × 10^8^	3.7 × 10^6^	2.9 × 10^6^	8.5 × 10^4^

The table shows the virus load expressed as TCID50 equivalents in lung sections of pigs euthanized post inoculation day (PID) 4 measured by quantitative real time RT-PCR detecting the matrix gene. Group 1 pigs were inoculated PID 0 with A/swine/Denmark/19126/1993 (H1N1) and group 2 pigs were inoculated PID 0 with A/swine/Denmark/10074/2004 (H1N2). Lu1: cranioventral part of the left cranial lobe; lu2: caudoventral part of the left cranial lobe; lu3: dorsal part of the left cranial lobe; lu4: ventral part of the right cranial lobe; lu5: middle part of the right cranial lobe; lu6: dorsal part of the right cranial lobe; lu7: middle part of the right middle lobe; lu8: middle part of the accessory lobe; lu9: middle part of the right caudal lobe.

## Discussion

The Danish “avian-like” H1N2 subtype variant was shown to harbour an HA gene of “avian-like” H1N1 SIV origin, thus differing significantly genetically and antigenic from the prevalent H1N2 viruses circulating in most of Europe which have a human-like HA gene. The phylogenetic analyses showed that the genes of the reassorted H1N2 virus had very high identity to contemporary H1N1 and H3N2 subtypes circulating in Danish pigs in 2003. Based on the sequence analysis, the Danish H1N2 subtypes apparently arose by two independent reassortment events. This is sustained by the full-genome sequencing analysis which revealed a closer relationship of the two analysed H1N2 isolates internal genes to Danish H1N1 and H3N2, respectively, than to each other. It is also interesting that all of the other sequenced Danish H1N2 viruses phylogenetically grouped together with A/swine/Denmark/13608/2004(H1N2), and that A/swine/Denmark/12687/2003(H1N2) formed a separate cluster. Interestingly, H1N2 viruses with high sequence similarity to the Danish H1N2 in all gene segments were recently reported from Sweden (A/swine/Sweden/1021/2009(H1N2) and A/swine/Sweden/9706/2010(H1N2)) and Italy (A/swine/Italy/58769/2009(H1N2)) [25-27]. The viruses from Sweden and Italy had the highest level of similarity to the A/swine/Denmark/13608/2004(H1N2) based on phylogeny and amino acid markers. The A/swine/Denmark/13608/2004(H1N2) had seven predicted glycosylation sites in the HA-protein compared to the A/swine/Denmark/12687/2003(H1N2) which had 4 sites. Glycosylation of the HA protein is important for virulence and antigenic masking of influenza A virus and could be the reason for the more successful establishment of the A/swine/Denmark/13608/2004(H1N2) virus [28,29]. At the receptor binding domain of the HA-protein, a different motif at position 225 was seen for the A/swine/Denmark/12687/2003(H1N2) compared to the other H1N2 viruses confirming a difference between the clusters. The H1N2 virus was detected in Denmark for the first time in 2003, approximately 6–7 years before it was found in Sweden. A publication from 2012 regarding Italian H1N2 SIV reports a retrospective finding of the “avian-like” H1N2 in samples from 2002 [27]. Before the discovery of the Danish H1N2 subtype, subtyping of SIV in Denmark was performed using HI-test. The HI-test only subtypes the HA of influenza A virus and the reassortant H1N2 virus may easily have been missed. Thus, due to the lack of systematic surveillance data in Europe at that time, the exact geographical origin of the new H1N2 subtype cannot be documented. Nevertheless, we speculate that the reassortment event happened between two Danish strains of H1N2 and H3N2 and subsequently spread to other European countries by export of living pigs. This hypothesis is supported by the full genome characterisation of the new reassorted viruses which showed that all gene segments were almost identical to genes of contemporary Danish H1N1 or H3N2 isolates. Furthermore, Denmark has a minimal import of live pigs from other countries which hamper introduction of new isolates. Accordingly, Denmark is free from the “human-like” H1N2 SIV which has been prevalent in central Europe for the last decades. The introduction of the virus into Sweden and Italy can be explained by the import of pigs from Denmark since pigs are not routinely tested for SIV in connection to export. Indeed, according to the National Association of Pig Breeders in Italy (ANAS), the import of living swine to Italy from Denmark is substantial. Taken together the Danish “avian-like” H1N2 subtype may be the first H1N2 subtype established within a pig population which possesses an HA of avian-like H1N1 origin.

The emergence of reassortant viruses in swine from concurrent circulating swine viruses occasionally occurs. Most European H1N2 viruses are closely related to A/swine/Scotland/4104/1994(H1N2) having an HA of human origin [9]. As an exception, A/swine/Italy/2064/1999(H1N2) has an HA gene related to the Danish H1N2 HA gene, and to concurrent Italian H1N1 and other European avian-like H1N1 viruses [9]. However, the NA gene of A/swine/Italy/2064/1999(H1N2) is closely related to NA genes of European H1N2 viruses, contrary to the Danish subtype which has an NA gene closer related to that of concurrent Danish H3N2 viruses. Thus, A/swine/Italy/2064/1999(H1N2) may be a reassortment of avian-like H1N1 and A/swine/Scotland/4104/94(H1N2)-like viruses. There have been no further reports on this virus, so this reassortant virus probably was unsuccessful in becoming established in the local and regional pig population. Recently, reassortments containing genes from the H1N1pdm09 virus have occurred *i.e.* an H1N2 with all genes from the pandemic virus except the N2 [21] and an H1N2 with HA and NA from the European swine H1N2 and the rest of the genes from the pandemic virus [22]. These findings further sustain the need for continuous SIV monitoring nationally and sharing of SIV surveillance data internationally.

Under the experimental conditions of the present study, the “avian-like” H1N2 subtype induced more severe macroscopic lung lesions compared to the older “avian-like” H1N1 subtype. The virus load in the lungs and the nasal excretions were slightly higher for the H1N2 infected pigs, however, the difference between groups was not statistically significant. In the pigs euthanized PID 4, virus was present in high amounts in most of the lung sections and did not show a predilection for any particular lung lobe, even though the macroscopic changes were more pronounced in the cranial parts. This is in accordance with findings of De Vleeschauwer *et al.*[30] who also were unable to demonstrate a particular pattern in virus titres in cranial and caudal lung lobes after an H1N1 infection in pigs. Initial reports from the field in 2004 claimed that the severity and duration of clinical signs elicited in response to the H1N2 virus was more severe compared with the H1N1 or H3N2 viruses, respectively (unpublished data). However, the results only provided a non-significant tendency for the H1N2 virus to be more virulent than the H1N1 virus. Apparently, the pigs elicited no clinical signs in response to experimental inoculation. The pigs were specific laboratory raised animals of high sanitary status free from the majority of pathogens known to cause disease in pigs [31]. They were kept in isolation without any contact to other animals eliminating the risk of exposure to new pathogens during the experiment. Furthermore, the pigs were not exposed to the same stress as pigs housed under field conditions and they had plenty of room and grooming material. This is probably the explanation for the lack of clinical signs even though the pigs clearly were infected as revealed by a slight rise in body temperature 1–3 days after challenge, the development of specific SIV antibodies, and the induction of an acute phase protein response following both the primary and the secondary inoculations. These observations further sustain that the clinical consequences of swine influenza virus infections under field conditions are highly dependent on management and the general health status of the herds.

Influenza A virus was excreted nasally from the day after inoculation until approximately PID 7 in pigs from both infection groups following the primary inoculation. After the second inoculation at PID 28, no or very low levels of nasal virus excretion was detected and only for one day which firmly indicate that there is a high level of cross-protection between the subtypes. An antibody response was elicited after inoculation of the first virus isolate and the response was boosted when the pigs were re-inoculated with the second virus isolate. Haptoglobin and CRP responses to primary infection with H1N1 and H1N2 were very similar. In contrast, no SSA response to primary infection with H1N2 was seen. Pomorska-Mól *et al.*[32] showed data on the induction of pig acute phase proteins CRP, haptoglobin and SAA after single experimental infection with a Polish “human-like” H1N2 SIV subtype and found a rapid and substantial increase in CRP, haptoglobin, and SAA. In the present study sampling was not performed at PID 2 and 3, therefore an SAA response to the primary H1N2 infection might have been missed. The results, however, corroborate well the results of Pomorska-Mól *et al.* showing pronounced haptogobin and CRP responses at day 4 post infection with H1N2. Taken together, these data indicated that infection with either H1N1 or H1N2 induces a high level of cross-protection against infection with the other virus. This was expected since the HA of the two subtypes is of the same “avian-like” origin.

The duration of the nasal virus excretion is in accordance with other experimental studies on SIVs showing excretion for 4–7 days after infection [33-35]. The duration of virus excretion following the primary H1N1 and H1N2 inoculation was comparable; however, the amount of virus excreted was approximately six-fold higher for pigs infected with H1N2 compared to H1N1 infected pigs. The H1N2 subtype has been very successful in spreading and constitute now 20% of the circulating subtypes in Danish pigs (own unpublished observations). The reason for this is not known but it’s interesting that this virus has succeeded in spreading despite existing immunity against the H protein of the H1N1 subtypes that has been circulating in Denmark since the mid-1980s.

## Conclusion

In conclusion, the “avian-like” H1N2 subtype has been circulating in Denmark since 2003 and originated probably by reassortment between Danish strains of “avian-like” H1N1 and H3N2. The “avian-like” H1N2 differs from other H1N2 subtypes in most of Europe and USA. There are two clusters of the Danish H1N2 variant and one of them seems to have spread to countries like Sweden and Italy. The experimental study shows comparable infection dynamic between the “avian-like” H1N2 and “avian-like” H1N1 with a tendency of the H1N2 to have a slightly larger impact on lungs. There were no clear clinical sign except elevated body temperature under the experimental conditions. Systemic responses to both virus subtypes were recognised by a clear antibody and acute phase response and there were a clear cross-protection between the two subtypes of “avian-like” H1N2 and “avian-like” H1N1.

## Materials and methods

### Viruses

Viruses were propagated in either Madin-Darby Canine Kidney Epithelial Cells (MDCK) or primary swine kidney cell cultures. Virus isolation was achieved from routine diagnostic samples, nasal swabs or lung tissue samples, sent to the Danish National Veterinary Laboratory for routine diagnostic purposes. Details on all viruses included in the study are summarised in Table 1.

### Sequencing and phylogenetic analysis

Sequencing of the HA and NA genes was performed as previously described by Bragstad *et a*l. 2005 [36] and full genome sequencing of all 8 gene segments was performed as described in Bragstad *et al.* 2006 [37]. Full genome sequencing was performed on the following virus isolates: A/swine/Denmark/13608/2004(H1N2), A/swine/Denmark/12687/2003(H1N2), A/swine/Denmark/14348-9/2003(H3N2), and A/swine/Denmark/14348-3/2003(H1N1).

For phylogenetic analyses, gene sequences were aligned with MUSCLE using CLC DNA Workbench version 6.8.1. Neighbour Joining trees were calculated with a bootstrap value of 1000.

Amino acid sequences were deduced from nucleotide sequences using CLC DNA Workbench version 6.8.1 and aligned with MUSCLE. N-glycosylation sites were predicted with NetNGlyc 1.0 (CBS, DTU, Denmark).

### Experimental infection

Landrace/Yorkshire (LY) pigs, both males and females, two months of age and free from more than 30 known swine pathogens including the important respiratory pathogens porcine circovirus 2 (PCV2), porcine reproductive and respiratory syndrome virus (PRRSV), swine influenza virus (SIV), porcine respiratory coronavirus, *Actinobacillus pleuropneumonia* and mycoplasmas [31], were used for the experiment. The pigs were randomly divided into two groups of 6 pigs, denoted group 1 and group 2. Each group was housed in separate isolation units (biosafety level 3). The pigs in group 1 were inoculated intranasally in the left nostril on PID 0 with 4 ml of A/swine/Denmark/19126/1993 (H1N1) with a titer of 10^5.7^ tissue culture infectious dose 50% (TCID50) per ml. Two pigs were euthanized on PID 4. On PID 28, the remaining four pigs were re-inoculated with 4 ml of A/swine/Denmark/10074/2004 (H1N2) with a titer of 10^5.9^ TCID50 per ml and they were euthanized on PID 56. For the pigs in group 2, the experimental design was similar except that the pigs were inoculated on PID 0 with the H1N2 isolate and then on PID 28 with the H1N1 isolate. Euthanization was performed by intravenous injection of pentobarbiturate (50 mg/kg) followed by exsanguination by cutting the *arteria axillaris.*

Body temperature was measured rectally before further handling of the pigs on PID -3, 0–9, and 28–37. Clinical signs and well-being of the pigs was monitored twice daily during the experiment. Blood samples were taken on PID -3, 0, 7, 11, 14, 18, 22, 28, 35, 39, 42, 46, 50, and 56 from *vena jugulars* from all pigs and from animals when euthanized on PID 4. Blood samples obtained on PID 0 and 28 were collected prior to inoculation. The blood samples were left on ice to coagulate for 15 minutes and centrifuged at 3500 rpm for 10 minutes at 4°C. Sera were transferred to new vials and stored at -80°C.

Nasal swab samples were collected on PID -3, 0–9, 11, 14, 18, 22, 28–37, 39, 42, 46, 50, and 56 from the right nostril. The swabs were placed in 1 ml phosphate buffered saline (PBS) (pH 7.5) and stored at -80°C until analysis. Nasal swabs taken on PID 0 and 28 were collected prior to inoculation. At post mortem, samples were collected and frozen at -80°C from the following nine parts of the lung, lu1: cranioventral part of the left cranial lobe; lu2: caudoventral part of the left cranial lobe; lu3: dorsal part of the left cranial lobe; lu4: ventral part of the right cranial lobe; lu5: middle part of the right cranial lobe; lu6: dorsal part of the right cranial lobe; lu7: middle part of the right middle lobe; lu8: middle part of the accessory lobe; and lu9: middle part of the right caudal lobe.

The study was carried out in strict accordance with the Danish legislation on animal experiments (LBK nr 1306 – 23/11/2007) and EU regulations on the use of laboratory animals for research.

### Acute phase proteins

Haptoglobin concentrations were determined by a sandwich ELISA as described previously [38]. The detection limit was 66 μg/ml.

A commercially available sandwich ELISA assay (Phase SAA assay, Tridelta Development Ltd., Kildare, Ireland) was used for determination of Serum Amyloid A (SAA) concentrations. This assay was based on anti-human monoclonal antibodies in a sandwich set-up as originally described by McDonald *et al.*[39]. The detection limit of the assay was 125 μg/ml (porcine SAA equivalents).

C – Reactive Protein (CRP) was analyzed by a sandwich type ELISA using dendrimer-coupled cytidine diphosphocholine (a CRP-binding ligand) in the coating layer as previously described [40] employing polyclonal rabbit anti-human antibodies with cross-reactivity towards porcine CRP followed by peroxidase-conjugated goat anti rabbit antibody for detection (both antibodies from DAKO, Glostrup, Denmark). The cross-reactivity of the anti human CRP antibody with porcine CRP was demonstrated previously [41]. The detection limit was 1416 ng/mL (human equivalents).

### Antibody detection

Antibodies were detected with the Hemagglutination Inhibition (HI) – test. Serum samples were pre-treated as follows: 100 μl of serum was inactivated at 56°C for 30 minutes. 400 μl of a 25% kaolin suspension in PBS was added and incubated for 20 minutes at room temperature and regularly shaken. The samples were centrifuged at 3000 rpm for 10 min, the supernatant mixed with 60 μl of packed chicken erythrocytes and incubated with the erythrocytes in a water bath at 37°C for 1 hour and regularly shaken. Finally, the samples were centrifuged at 3000 rpm for 10 min and the supernatant was used in the HI-test.

In the HI-test, 25 μl PBS was added to each well of a microtitre plate, then 25 μl of the sample was added and diluted two-fold. Then 25 μl of the antigen (4 HA units) were added to each well. The plate was shaken for 10 sec and incubated for 1 hour at room temperature. After incubation, 25 μl of 0.6% chicken erythrocyte dilution was added to the wells, shaken for 10 sec and incubated for 30 minutes at room temperature. The HI-titre of the serum samples was determined as the highest dilution showing complete inhibition of agglutination.

Chicken erythrocytes were used as standard blood cells. During testing it was noted that the H1N2 isolate used for inoculation (A/swine/Denmark/10074/2004 (H1N2)) did not have the ability to agglutinate chicken erythrocytes. Instead guinea pig erythrocytes were used for this isolate. The procedure for the HI-test using guinea pig erythrocytes was similar except that a 1% suspension of erythrocytes in PBS with 0.005% gelatine was used.

### RNA purification

RNA was purified from nasal swabs and lung tissue samples with RNeasy Minikit (Qiagen, GmbH, Germany). 200 μl nasal swab sample was lysed in 400 μl RLT-buffer containing 1% ß-mercaptoethanol while 30 mg of lung tissue were homogenized and lysed with 600 μl RLT-buffer containing ß-mercaptoethanol by bead-beating. After lysis, the RNA was purified according to the kit instructions. RNA was stored at -80°C until analysis. RNA was extracted from all nasal swabs and lung tissue samples and tested by a real time RT-PCR screenings assay. All positive samples were further tested in a quantitative real time RT-PCR assay.

### Real time RT-PCR screening assay

A previously designed general influenza A virus RT-PCR assay with specific primers for a conserved region of the nucleoprotein (NP) gene described by Munch *et al.*[42] was modified into a real time assay using SYBR green chemistry detection. This assay was used to screen the nasal swabs and tissue samples for influenza A virus specific RNA. The real time RT-PCR was performed in a Rotor-Gene 3000 machine (Corbett Research, Australia) in a total volume of 25 μl using the Qiagen OneStep RT-PCR kit (Qiagen, GmbH, Germany) with 2 μl of extracted RNA, 0.6 μM of each primer (Forward Primer MMU39 and Reverse Primer MMU19), and 6.25× SYBR green (Invitrogen SYBR® Green I Nucleic acid stain 10000×). The amplification temperature profile was 50°C for 30 min for reverse transcription followed by 95°C for 15 min and 45 cycles of 94°C for 20 s, 60°C for 20 s and 72°C for 30 s. This was immediately followed by a melting point analysis: 50°C for 60 s, ramping from 50°C to 99°C in increments of 1°C and holding for 15 s at each step. The fluorescence signal was measured at 72°C during each PCR cycle and at each temperature increment step during the melting analysis. All fluorescence measurements were analysed with the Rotor-Gene Software Version 6.0. with non template control (NTC) threshold set at 10% and the normalised fluorescence threshold limit set at 0.05 for cycle threshold (Ct values) determination.

### Relative quantification by real time RT-PCR

For quantification of virus load in nasal swabs and in lung tissue a quantitative real time RT-PCR assay based on Primer Probe Energy Transfer technology (PriProET) targeting a conserved region of the matrix gene was used [30,43]. The real time RT-PCR was performed in a Rotor-Gene 3000 machine (Corbett Research, Australia) with a total reaction volume of 25 μl using the RNA Ultrasense™ One-Step Quantitative RT-PCR System (Invitrogen), 5 μl of extracted RNA, 0.4 μM of forward primer, 1.0 μM of FAM-labelled reverse primer, and 1.0 μM of Cy5-labelled probe. The amplification temperature profile was 50°C for 30 min for reverse transcription followed by 95°C for 5 min and 45 cycles of 95°C for 15 s, 55°C for 30 s and 72°C for 20 s and finally by 72°C for 5 min. This was immediately followed by a melting point analysis: 95°C for 30 s, ramping from 40°C to 99°C in increments of 1°C and holding for 30 s at each step. The fluorescence signal was measured at 55°C during each PCR cycle and at each temperature increment step during the melting analysis. Signals were generated by excitation of the donor fluorophor (FAM) followed by collection of emission spectra from the acceptor fluorophor (Cy5). All fluorescence measurements were analysed with the Rotor-Gene Software Version 6.0. In each real time RT-PCR run, RNA purified from cell cultured A/swine/Denmark/19126/1993 (H1N1) with 10^3.8^ TCID50 per ml was used as positive control and RNase free water as negative control. The NTC threshold was set at 10% and the normalised fluorescence threshold limit at 0.02 for cycle threshold (Ct) value determination. A standard curve for the real time RT-PCR assay was obtained using a ten-fold dilution series made from an initial concentration of 10^7.8^ TCID50 per ml of the virus A/swine/Denmark/19126/1993 (H1N1). Ct-values from samples were calibrated towards the standard curve to obtain a relative quantification of TCID50 equivalents. The efficiency of the PCR was 92% and the range of quantification spanned 6 log_10_ dilutions from dilution 10^-1^ to 10^-7^.

### Statistical analysis

To determine whether there was a statistical significant difference of nasal virus excretion, virus load in lungs, and acute phase proteins between the two groups of pigs an un-paired *t*-test with a 95% confidence interval was applied [44].

## Competing interests

The authors have no competing interests.

## Authors’ contributions

RT participated in the conception and design of the experimental study, performed post mortem investigation, RT-PCR analysis, HI-test, phylogeny and drafted the manuscript. KB did the sequencing and sequence analysis. LEL participated in the conception and design of the experimental study, and performed post mortem investigation. JN conducted the experimental infection study, sampling, and post mortem investigation. AB obtained the clinical samples and organized sample processing. PH performed acute phase protein analysis. AF participated in planning and design of the sequencing and phylogeny. BV participated in the conception and design of the experimental study. CKH did sequence analysis, phylogeny, and drafted the manuscript. All authors contributed to planning of the work, interpretation of the findings and revised the manuscript. All authors approved the final manuscript.

## References

[B1] Alexander DJ An overview of the epidemiology of avian influenza Vaccine 2007 10 5637 5644 10.1016/j.vaccine.2006.10.051 17126960

[B2] Fouchier RAM Munster V Wallensten A Bestebroer TM Herfst S Smith D Characterization of a novel influenza A Virus Hemagglutinin Subtype (H16) Obtained from Black-Headed Gulls J Virol 2005 10 2814 2822 10.1128/JVI.79.5.2814-2822.2005 15709000 PMC548452

[B3] Webster RG Bean WJ Gorman OT Chambers TM Kawaoka Y Evolution and ecology of influenza A viruses Microbiol Rev 1992 10 152 179 1579108 10.1128/mr.56.1.152-179.1992 PMC372859

[B4] Tong S Li Y Rivailler P Conrardy C Castillo DAA Chen LM A distinct lineage of influenza A virus from bats Proc Natl Acad Sci 2012 10 4269 4274 22371588 10.1073/pnas.1116200109 PMC3306675

[B5] Brown IH Ludwig S Olsen CW Hannoun C Scholtissek C Hinshaw VS Harris PA McCauley JW Strong I Alexander DJ Antigenic and genetic analyses of H 1 N 1 influenza A viruses from European pigs J Gen Virol 1997 10 553 562 9049404 10.1099/0022-1317-78-3-553

[B6] Castrucci MR Donatelli I Sidoli L Barigazzi G Kawaoka Y Webster RG Genetic Reassortment between Avian and Human Influenza A Viruses in Italian Pigs Virology 1993 10 503 506 10.1006/viro.1993.1155 8438586

[B7] Brown IH Chakraverty P Harris PA Alexander DJ Disease outbreaks in pigs in Great Britain due to an influenza A virus of H1N2 subtype Veterinary Record 1995 10 328 329 10.1136/vr.136.13.328 7541591

[B8] Brown IH Harris PA McCauley JW Alexander DJ Multiple genetic reassortment of avian and human influenza A viruses in European pigs, resulting in the emergence of an H1N2 virus of novel genotype J Gen Virol 1998 10 2947 2955 9880008 10.1099/0022-1317-79-12-2947

[B9] Marozin S Gregory V Cameron K Bennett M Valette M Aymard M Antigenic and genetic diversity among swine influenza A H1N1 and H1N2 viruses in Europe J Gen Virol 2002 10 735 745 11907321 10.1099/0022-1317-83-4-735

[B10] Van Reeth K Brown IH Pensaert M Isolations of H1N2 influenza A virus from pigs in Belgium Veterinary Record 2000 10 588 589 10.1136/vr.146.20.588 10839238

[B11] Schrader C Süss J Genetic Characterization of a Porcine H1N2 Influenza Virus Strain Isolated in Germany Intervirology 2003 10 66 70 10.1159/000068124 12566701

[B12] Maldonado J Van Reeth K Riera P Sitja M Saubi N Espuna E Evidence of the concurrent circulation of H1N2, H1N1 and H3N2 influenza A viruses in densely populated pig areas in Spain Vet J 2006 10 377 381 10.1016/j.tvjl.2005.04.014 15914047

[B13] Zell R Motzke S Krumbholz A Wutzler P Herwig V Durrwald R Novel reassortant of swine influenza H1N2 virus in Germany J Gen Virol 2008 10 271 276 10.1099/vir.0.83338-0 18089751

[B14] Vincent A Ma W Lager K Gramer M Richt J Janke B Characterization of a newly emerged genetic cluster of H1N1 and H1N2 swine influenza virus in the United States Virus genes 2009 10 176 185 10.1007/s11262-009-0386-6 19597980

[B15] Chutinimitkul S Thippamom N Damrongwatanapokin S Payungporn S Thanawongnuwech R Amonsin A Genetic characterization of H1N1, H1N2 and H3N2 swine influenza virus in Thailand Arch Virol 2008 10 1049 1056 10.1007/s00705-008-0097-7 18458812

[B16] Ito T Kawaoka Y Vines A Ishikawa H Asai T Kida H Continued circulation of reassortant H1N2 influenza viruses in pigs in Japan Arch Virol 1998 10 1773 1782 10.1007/s007050050415 9787660

[B17] Jung K Chae C Phylogenetic analysis of an H1N2 influenza A virus isolated from a pig in Korea Arch Virol 2004 10 1415 1422 15221541 10.1007/s00705-004-0324-9

[B18] Karasin AI Landgraf J Swenson S Erickson G Goyal S Woodruff M Scherba G Anderson G Olsen CW Genetic characterization of H1N2 influenza A viruses isolated from pigs throughout the United States J Clin Microbiol 2002 10 1073 1079 10.1128/JCM.40.3.1073-1079.2002 11880444 PMC120269

[B19] Qi X Lu CP Genetic characterization of novel reassortant H1N2 influenza A viruses isolated from pigs in southeastern China Arch Virol 2006 10 2289 2299 10.1007/s00705-006-0796-x 16755371 PMC7087176

[B20] Shope RE Swine Influenza III. Filtration Experiments and Etiology J Exp Med 1931 10 373 385 10.1084/jem.54.3.373 19869924 PMC2132000

[B21] Moreno A Di Trani L Faccini S Vaccari G Nigrelli D Boniotti MB Novel H1N2 swine influenza reassortant strain in pigs derived from the pandemic H1N1/2009 virus Vet Microbiol 2011 10 472 477 10.1016/j.vetmic.2010.12.011 21208754

[B22] Howard W Essen SC Strugnell BW Russell C Barrass L Reid SM Reassortant pandemic (H1N1) 2009 virus in pigs, United Kingdom Emerg Infect Dis 2011 10 1049 1052 21749767 10.3201/eid1706.101886 PMC3358214

[B23] Gourreau JM Kaiser C Valette M Douglas AR Labie J Aymard M Isolation of two H1N2 influenza viruses from swine in France Arch Virol 1994 10 365 382 10.1007/BF01310021 7979974

[B24] Bøtner A Svineinfluenza Veterinær Information 1994 10 24 2716978821

[B25] Balint A Metreveli G Widen F Zohari S Berg M Isaksson M The first Swedish H1N2 swine influenza virus isolate represents an uncommon reassortant Virol J 2009 10 180 10.1186/1743-422X-6-180 19863790 PMC2774320

[B26] Metreveli G Emmoth E Zohari S Bálint A Widén F Muradrasoli S Comparison of two H1N2 swine influenza A viruses from disease outbreaks in pigs in Sweden during 2009 and 2010 Virus Genes 2011 10 236 244 10.1007/s11262-011-0571-2 21253862

[B27] Moreno A Chiapponi C Boniotti MB Sozzi E Foni E Barbieri I Genomic characterization of H1N2 swine influenza viruses in Italy Vet Microbiol 2012 10 265 276 10.1016/j.vetmic.2011.11.004 22112856

[B28] Deshpande KL Fried VA Ando M Webster RG Glycosylation affects cleavage of an H5N2 influenza virus hemagglutinin and regulates virulence Proc Natl Acad Sci 1987 10 36 40 10.1073/pnas.84.1.36 3467357 PMC304136

[B29] Skehel JJ Stevens DJ Daniels RS Douglas AR Knossow M Wilson IA A carbohydrate side chain on hemagglutinins of Hong Kong influenza viruses inhibits recognition by a monoclonal antibody Proc Natl Acad Sci 1984 10 1779 1783 10.1073/pnas.81.6.1779 6584912 PMC345004

[B30] De Vleeschauwer A Atanasova K Van Borm S van den Berg T Rasmussen TB Uttenthal A Comparative Pathogenesis of an Avian H5N2 and a Swine H1N1 Influenza Virus in Pigs PLoS ONE 2009 10 e6662 doi:10.1371/journal.pone.0006662 10.1371/journal.pone.0006662 19684857 PMC2722722

[B31] Ladekjær-Mikkelsen A-S Nielsen J Stadejek T Storgaard T Krakowka S Ellis J Reproduction of postweaning multisystemic wasting syndrome (PMWS) in immunostimulated and non-immunostimulated 3-week-old piglets experimentally infected with porcine circovirus type 2 (PCV2) Vet Microbiol 2002 10 97 114 10.1016/S0378-1135(02)00174-8 12243888 PMC7117141

[B32] Pomorska-Mól M Markowska-Daniel I Kwit K Immune and acute phase response in pigs experimentally infected with H1N2 swine influenza virus FEMS Immunol Med Microbiol 2012 10 334 342 10.1111/j.1574-695X.2012.01026.x 22924885

[B33] Van Reeth K Labarque G Pensaert M Serological Profiles after Consecutive Experimental Infections of pigs with European H1N1, H3N2, and H1N2 Swine Influenza Viruses Viral Immunol 2006 10 373 382 10.1089/vim.2006.19.373 16987057

[B34] Van Reeth K Gregory V Hay A Pensaert M Protection against a European H1N2 swine influenza virus in pigs previously infected with H1N1 and/or H3N2 subtypes Vaccine 2003 10 1375 1381 10.1016/S0264-410X(02)00688-6 12615433

[B35] Hermann JR Brockmeier SL Yoon K-J Zimmerman JJ Detection of respiratory pathogens in air samples from acutely infected pigs Can J Vet Res 2008 10 367 370 18783027 PMC2442681

[B36] Bragstad K Jørgensen PH Handberg KJ Mellergaard S Corbet S Fomsgaard A New avian influenza A virus subtype combination H5N7 identified in Danish mallard ducks Virus Res 2005 10 181 190 10.1016/j.virusres.2004.12.004 15763149

[B37] Bragstad K Jørgensen PH Handberg KJ Fomsgaard A An emerging avian influenza A virus H5N7 is a genetic reassortant of highly pathogenic genes Vaccine 2006 10 6736 6741 10.1016/j.vaccine.2006.05.056 16814904

[B38] Sorensen NS Tegtmeier C Andresen LO Piñeiro M Toussaint MJ Campbell FM The porcine acute phase protein response to acute clinical and subclinical experimental infection with *Streptococcus suis* Vet Immunol Immunopathol 2006 10 157 168 10.1016/j.vetimm.2006.04.008 16774789

[B39] McDonald TL Weber A Smith JW A monoclonal antibody sandwich immunoassay for serum amyloid A (SAA) protein J Immunol Methods 1991 10 149 155 10.1016/0022-1759(91)90081-P 1720442

[B40] Heegaard PMH Pedersen HG Jensen AL Boas U A robust quantitative solid phase immunoassay for the acute phase protein C-reactive protein (CRP) based on cytidine 5′-diphosphocholine coupled dendrimers J Immunol Meth 2009 10 112 118 10.1016/j.jim.2009.02.002 19236874

[B41] Heegaard PMH Klausen J Nielsen JP Gonzáles-Ramón N Piñeiro M Lampreave F The porcine acute phase response to infection with *Actinobacillus pleuropneumoniae*. Haptoglobin, C-reactive protein, major acute phase protein and serum amyloid A protein are sensitive indicators of inflammation Comp Biochem Physiol 1998 10 365 373 10.1016/S0305-0491(97)00362-3 9629669

[B42] Munch M Nielsen LP Handberg KJ Jorgensen PH Detection and subtyping (H5 and H7) of avian type A influenza virus by reverse transcription-PCR and PCR-ELISA Arch Virol 2001 10 87 97 10.1007/s007050170193 11266220

[B43] Fouchier RAM Bestebroer TM Herfst S Van Der Kemp L Rimmelzwaan GF Osterhaus ADME Detection of Influenza A Viruses from Different Species by PCR Amplification of Conserved Sequences in the Matrix Gene J Clin Microbiol 2000 10 4096 4101 11060074 10.1128/jcm.38.11.4096-4101.2000 PMC87547

[B44] Altman DG Practical statistics for medical research 1999 1 Boca Raton, FL: Chapman & Hall/CRC press

